# Multi-Dimensional Dataset of Open Data and Satellite Images for Characterization of Food Security and Nutrition

**DOI:** 10.3389/fnut.2021.796082

**Published:** 2022-01-27

**Authors:** David S. Restrepo, Luis E. Pérez, Diego M. López, Rubiel Vargas-Cañas, Juan Sebastian Osorio-Valencia

**Affiliations:** ^1^Telematics Department, Telematics Engineering Research Group, Universidad del Cauca, Popayán, Colombia; ^2^Physics Department, Dynamic Systems, Instrumentation and Control Research Group, Universidad del Cauca, Popayán, Colombia; ^3^Department of Global Health, University of Washington, Seattle, WA, United States

**Keywords:** data mining, food security, machine learning, remote sensing, satellite imagery, dataset

## Abstract

**Background:**

Nutrition is one of the main factors affecting the development and quality of life of a person. From a public health perspective, food security is an essential social determinant for promoting healthy nutrition. Food security embraces four dimensions: physical availability of food, economic and physical access to food, food utilization, and the sustainability of the dimensions above. Integrally addressing the four dimensions is vital. Surprisingly most of the works focused on a single dimension of food security: the physical availability of food.

**Objective:**

The paper proposes a multi-dimensional dataset of open data and satellite images to characterize food security in the department of Cauca, Colombia.

**Methods:**

The food security dataset integrates multiple open data sources; therefore, the Cross-Industry Standard Process for Data Mining methodology was used to guide the construction of the dataset. It includes sources such as population and agricultural census, nutrition surveys, and satellite images.

**Results:**

An open multidimensional dataset for the Department of Cauca with 926 attributes and 9 rows (each row representing a Municipality) from multiple sources in Colombia, is configured. Then, machine learning models were used to characterize food security and nutrition in the Cauca Department. As a result, The Food security index calculated for Cauca using a linear regression model (Mean Absolute Error of 0.391) is 57.444 in a range between 0 and 100, with 100 the best score. Also, an approach for extracting four features (Agriculture, Habitation, Road, Water) of satellite images were tested with the ResNet50 model trained from scratch, having the best performance with a macro-accuracy, macro-precision, macro-recall, and macro-F1-score of 91.7, 86.2, 66.91, and 74.92%, respectively.

**Conclusion:**

It shows how the CRISP-DM methodology can be used to create an open public health data repository. Furthermore, this methodology could be generalized to other types of problems requiring the creation of a dataset. In addition, the use of satellite images presents an alternative for places where data collection is challenging. The model and methodology proposed based on open data become a low-cost and effective solution that could be used by decision-makers, especially in developing countries, to support food security planning.

## Introduction

In 2015, United Nations member states adopted the Sustainable Development Goals (SDG), a commitment to accomplishing before 2030 a set of universal actions to end poverty, protect the planet, and promote peace and prosperity in the world (1). They placed a high priority on food security and nutrition (SDG). The target for 2030 included, among others, ending hunger and all forms of malnutrition; ensuring access to safe, nutritious, and sufficient food all year round; doubling the agricultural productivity and incomes of small-scale food producers and ensuring sustainable food production systems; and implementing resilient agricultural practices that increase productivity and production. Considering the above, establishing food security actions and measuring their impact is critical for decision-making in a country or specific region like a department or municipality.

According to the Food and Agriculture of the United Nations (FAO), food security encompasses four dimensions: physical availability of food economic and physical access to food, food utilization, and the sustainability of the dimensionsmentioned above (2). These dimensions must be taken into account to calculate a comprehensive food security index that allows characterizing and comparing the food security levels of a country or geographic region. Some works have been proposed around creating a food security index in which the four dimensions proposed by FAO are integrated. Among them, the one that stands out the most is the Global Food Security Index (GFSI), introduced by the Economist Intelligence Unit (EIU) (3). While this index meets the dimensions and has proven to be accurate (4), it is an index that is difficult to calculate mainly due to the large amount of data required and the costs involved in collecting this data. Therefore, in most cases, the GFSI is applied only at the country level and not at the regional level.

There are other alternatives for calculating a food security index without requiring a large amount of data. Some examples are the Global Hunger Index (GHI) (5) or The Food Insecurity Experience Scale (FIES) from FAO (6). However, these indices do not meet all the dimensions described by GFSI. They focus on nutritional and physical access issues, which gives us an incomplete perspective on the problem. The challenge then for governments is to have comprehensive and complete sources of information to characterize the food security situation at the national, regional, or local level to support decision-making and achieve the SDGs.

According to the information described in the Colombian National Survey of Nutritional Situation document (7), food insecurity in Colombia per household was 54.2%, which means that at least one out of every two households does not guarantee food security. In households with food insecurity, 13.8% corresponds to moderate food insecurity, and 8.5% corresponds to severe food insecurity. This situation is aggravated in the rural area, where it represents 64.1%. According to GFSI data, Colombia's food security index corresponds to 63.2 points (this value indicates how favorable the food security environment is in the country). This result is the most complete that can be found at the country level. However, Colombia is a large country with multiple landscapes and economies, with food security affected by them. Therefore, for decision-making at the departmental or municipal level, it is necessary to calculate an adapted and specific food security index. However, no data is available at this level.

For the Department of Cauca, one of the more vulnerable and underserved regions in Colombia; collecting the data necessary to calculate a complete food security index is a highly complex and expensive process due to the difficulties in accessing some locations. Given this problem raises the possibility of using satellite data to cover remote areas through images (8), in addition to other meteorological data and combine these with different data obtained from other sources such as nutrition or agriculture surveys, censuses, or health reports, so that a completely open data multidimensional dataset that allows calculating the GFSI for this department can be obtained.

Machine Learning models (ML) can be potentially used for the calculation of food security. Nevertheless, current models are focused on analyzing the first FAO's dimension of physical availability of food through the prediction of crop yield (9–24), and are not used to calculate an index itself. On the other hand, other techniques such as GHI and FIES used for calculating a food security index at a regional level mainly focus on nutritional or economic and physical access aspects (25). As a result, it leaves out the four dimensions described by FAO.

This paper aims to build a multi-dimensional dataset of open data and satellite images to characterize food security in the Department of Cauca, Colombia. The dataset is structured according to the four-dimensional model of food security proposed by FAO. An ML-based model that calculates the GFSI for the Department of Cauca is trained and tested with the GFSI data to validate the dataset.

## Materials and Methods

There are many methodologies and indicators to measure food security. They can broadly be classified based on the analysis of (a) primary data sources (expert opinion and community perspectives) and (b) secondary data (26). In building a comprehensive dataset, using open and secondary data sources with varied nature and format, and large volumes of information, it was decided to follow the Cross-Industry Standard Process for Data Mining methodology (CRISP-DM) (27). CRISP-DM is the most widely used methodology in data mining projects analyzing large datasets. This methodology consists of 6 phases (Business Understanding, Data Understanding, Data Preparation, Modeling, Evaluation, and Deployment). In this work, the first five phases of the methodology were addressed. The last phase, deployment, is outside the scope of this paper.

Following the methodology we expected to obtain a dataset that addresses the four dimensions described by FAO. Additionally, the quality of the database is validated using an ML model to calculate the GFSI in some municipalities of Cauca. The phases are explained below.

### Business Understanding

In this phase, we reviewed the existing literature to determine the causes of food security and nutritional problems, as well as possible consequences and existing measures. The results were categorized into the four dimensions described by FAO:

- Physical availability of food. This dimension includes the quantity of food offered, which comprises food production, stocks, and net trade.
- Economic and physical access to food. In addition to having physical availability, it must also be guaranteed that the population can acquire these foods. To obtain food, they must have economic resources and easy access (i.e., roads, avenues, routes).
- Food utilization. Having access to food is not always enough. Aspects such as a healthy diet, optimal food preparation, a good distribution of nutrients, and quality food must also be considered.
- The sustainability of the dimensions mentioned above is one factor that makes measuring food security so complex. In addition, environmental, political, or economic variables are considered in this dimension and may also influence food security indices over time.

The GFSI is the selected food security index as it is the only one that covers the four dimensions described by FAO. The challenge would be in the data collection required for its calculation. Due to its complexity, it is an index calculated at a country level but not for specific regions like departments or municipalities. Then the use of a methodology for data mining is necessary to create a dataset and the respective ML model. It confirms the need to use CRISP-DM as the methodology for this research.

### Data Understanding

Once we understood the problem, we started with the data collection. The data was structured according to the fields provided by the GFSI and the FAO dimensions For example, in the case of Cauca, the data collected were:

- Satellite Imagery: the Google Earth Engine (GEE) platform (28) offers a dataset of Landsat-8 satellite imagery from April 2013 to the present. This satellite provides an image with an approximate frequency of once every 16 days and has different bands where the B4, B3, and B2 bands are the Red, Green, and Blue bands, respectively. These bands can be used to build an RGB image with a resolution of 30 m/px, and the B8 is the Panchromatic band with a resolution of 15 m/px and can be used to increase the resolution of the RGB image. These data were collected due to the potential for obtaining information on crop and water sources (physical availability), roads (economic and physical access), and temporal scale (sustainability of the dimensions over time).
- Nutrition data: Colombia's nutrition data are obtained from the National Survey of Nutritional Situation (ENSIN) (29). This is a survey carried out every 5 years by a public entity of the Colombian government to classify the nutritional situation by departments and municipalities. It has information on economic income and sociodemographic data belonging to the economic and physical access dimension, and the food consumed and the vitamins of a person (food utilization). This is the most comprehensive survey in the country related to nutrition and food security. The available data correspond to the year 2015.
- Agricultural Census: the agricultural census data come from the National Statistics Office (DANE) (30), and correspond to the 2014 census. In this data, farmers are asked questions about the conditions of some crops, access to financing, infrastructure, and planted area. These data are part of the dimension of the physical availability of food. These surveys are carried out at the municipality level by department.
- Health Records: health data is usually the most complex data to obtain due to the resources and tools necessary to get it and the limitations of information privacy. However, it is possible to access public records that are part of the National Public Health Surveillance System (SIVIGILA) (31). From this system, information about the weekly admissions to hospitals can be obtained. In this dataset, data on acute malnutrition in children under 5 years of age can be obtained from January 2016 to the present. Also, data on mortality due to malnutrition from January 2013 to the present. These data would allow us to analyze sustainability.
- Meteorological Data: within the category of meteorological data, many types of data from different sources are relevant to this study, such as temperature, soil type, precipitation, soil maps, humidity, etc. However, due to the complexity of processing these data, we only use the temperature and precipitation, that are part of the physical availability of food dimension. Therefore, two primary data sources were considered: WorldClim (32) and Google Earth Engine–GEE. The first provides pre-processed information on monthly temperature and precipitation data from 1960 to 2018, while in the second, this temporal resolution depends on the data source.

### Data Preparation

In this phase, data preprocessing was done to have it ready for developing the model. This phase includes data selection, cleaning, changing formats, creating new variables, and merging. In this case, the data preparation was divided into two sections according to the two existing data modalities, satellite images, and metadata.

#### Data Preparación for Satellite Imagery

##### Image Selection

Because the proposal of this section is only an alternative to the lack of data and in this case it is not the data used for the calculation of the GFSI, images were used only for the capital of the department of Cauca (Popayán). Then, the GEE platform was used to crop the images of the selected satellite. For this case, Landsat-8 was used, and the limits of each image were defined with the shapefile of the municipality.

##### Cloud and Shadow Removal

**Figure 1:**
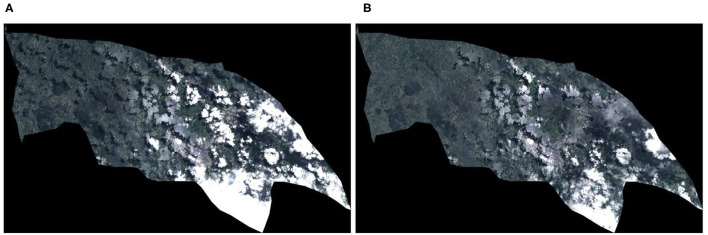
Resulting image choosing different percentages in the composite. **(A)** Landsat satellite composite image of the municipality of Popayán using images collected during 2 months. **(B)** Landsat satellite composite image of the municipality of Popayán using images collected during 4 months.

**Figure 2:**
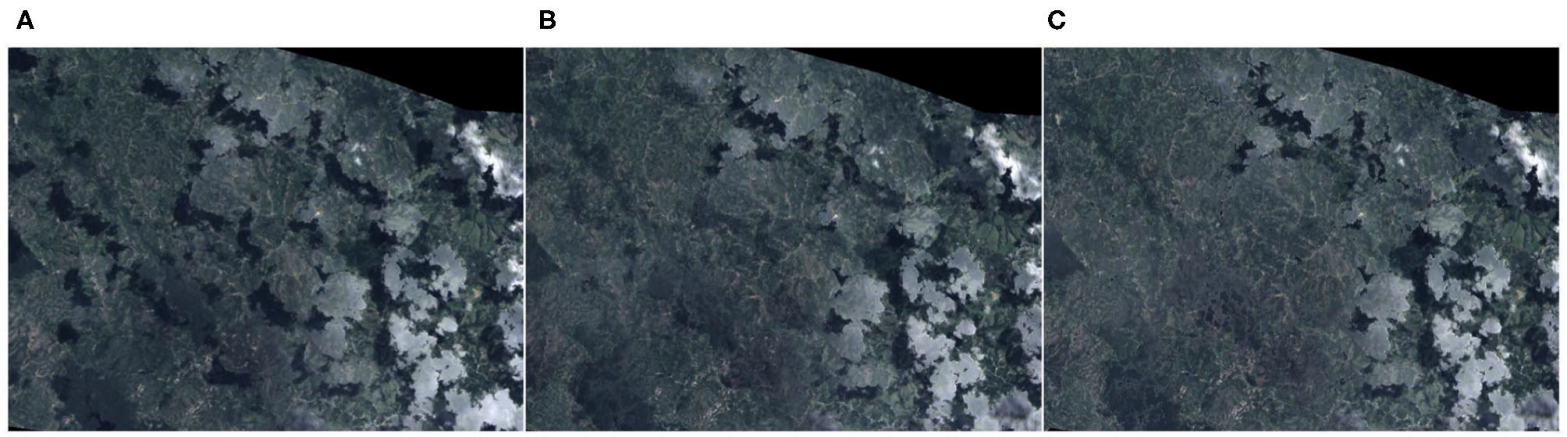
Resulting image choosing different percentages in the composite. **(A)** Composite of landsat images using the pixel value in the percentile 20th. **(B)** Composite of landsat images using the pixel value in the percentile 50th. **(C)** Composite of landsat images using the pixel value in the percentile 75th.

Monthly images of the satellite were created trying to eliminate as many clouds and shadows as possible. To create the image, a composition was made of a series of images captured in an interval of 3 months in the future and 1 month in the past in the area to be studied in a given month. Such a long time interval was used to get more image samples and cover more areas without clouds or shadows. A smaller quantity of images resulted in an image of poorer quality, as seen in Figure 1. The method used here calculated the percentage of clouds in each pixel and selected the pixels with the lowest rate of clouds. Among those pixels with the lowest ratio of clouds, the value was taken at the 75th percentile. The reason for using the 75th percentile value was that pixels with shadows correspond to the darkest values (lowest percentiles), as shown in Figure 2. Therefore, the pixels with the highest cloud index (the lightest) were eliminated.

##### Resolution Increase

**Figure 3:**
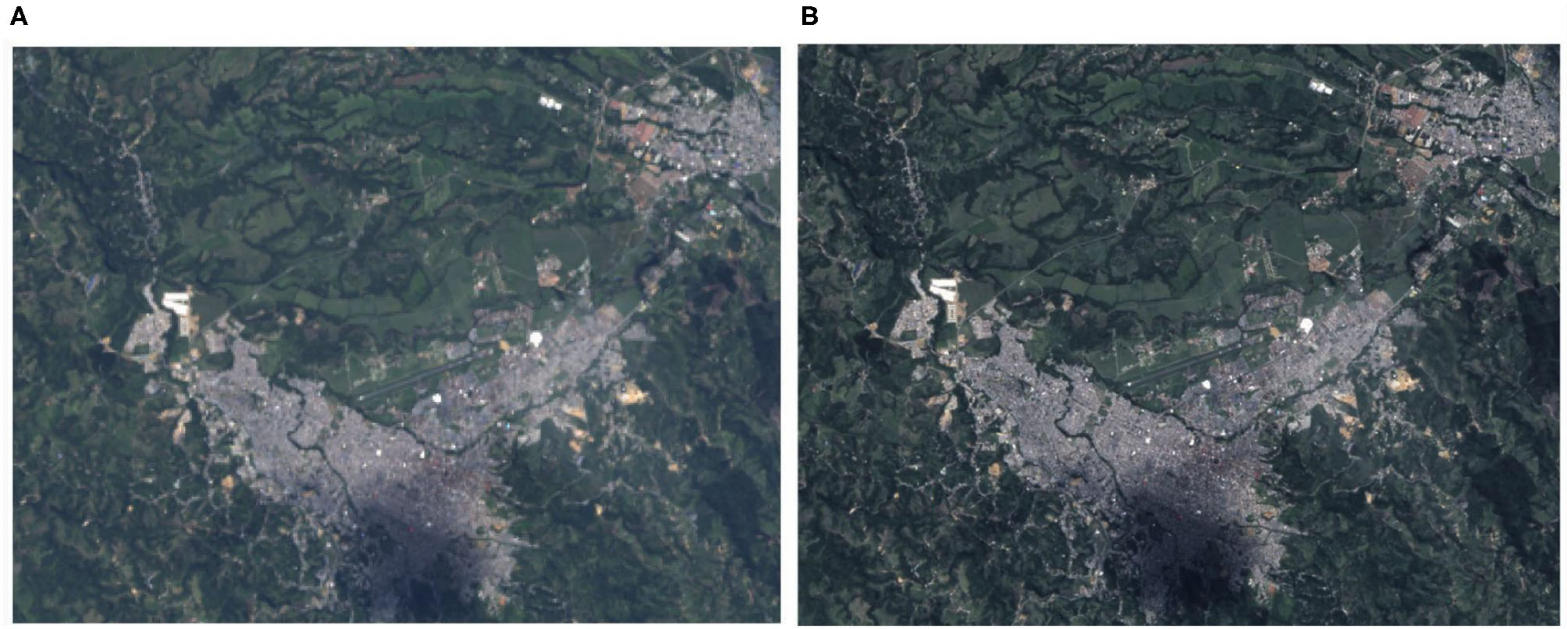
Increased resolution. **(A)** Landsat satellite image of the municipality of Popayán with the original RGB resolution of 30 m/px. **(B)** Landsat satellite image of the municipality of Popayán with the increased RGB resolution of 15 m/px.

The resolution of the images was increased in the bands B4, B3, and B2, corresponding to the colors red, green, and blue. To do this, a transformation of the image was made from RGB to HSV, and was operated with band 8 (Panchromatic) at 15 m/px. Finally, a reconversion from HSV to RGB is done again with the values scaled to 15 m/px, as shown in Figure 3.

##### Features Extraction

To use satellite images in conjunction with metadata from other sources, there are several techniques. We selected the extraction of features from the image due to its simplicity and interpretability. The characteristics extracted were agriculture, inhabitants, roads, and water. For this, deep learning models are trained to detect the presence of the four characteristics using the dataset “Planet: Understanding the Amazon from Space” available at Kaggle (33). The “Planet” dataset has 40,479 Images of 256 × 256 pixels, each image tagged with the features present in it. Once the images were selected, the dataset was randomly divided into two parts, 80% used in training and the test data with the remaining 20%. The training dataset was subdivided randomly into 90% for training and 10% for validation. The task was a multi-label classification in which models such as ResNet 50 (34), VGG 16 (35), and Visual Transformers (ViT) (36). The VGG 16 model was also trained using the pre-trained weights from ImageNet. To evaluate each model, precision, recall, and the f1-score were calculated to predict each class in the 20% of test data. The model selected as the best was the model with the highest f1-score (**Figure 8**) because it is the most balanced metric from those calculated.

The best model was a ResNet 50 model trained from scratch. The original architecture of ResNet 50 was modified to have as input a tensor of shape 256, 256, 3 because of the shape of the images in Planet dataset. The output was also modified to have a number of neurons as the number of features to predict, each neuron with a sigmoid activation function. The model was trained using batches of 32 images as input, each image with a random augmentation (flipped, shift between 0 and 15%, zoom between 0 and 40%, and rotation between 0 and 30%). The output was a matrix with batch size as the number of rows and each feature as a column indicating if there is presence of a given feature in the image (1) or not (0). To train the model the loss function used was binary cross entropy in conjunction with an Adam optimizer with 0.001 as learning rate, 0.9 as beta 1, 0.999 as beta 2, and 1e-07 as epsilon. The model was trained using early stop comparing the validation losses to avoid overfitting. If the validation losses increased during 5 consecutive epochs by a value >1e-3, the training would stop and return to the best weights.

**Figure 4:**
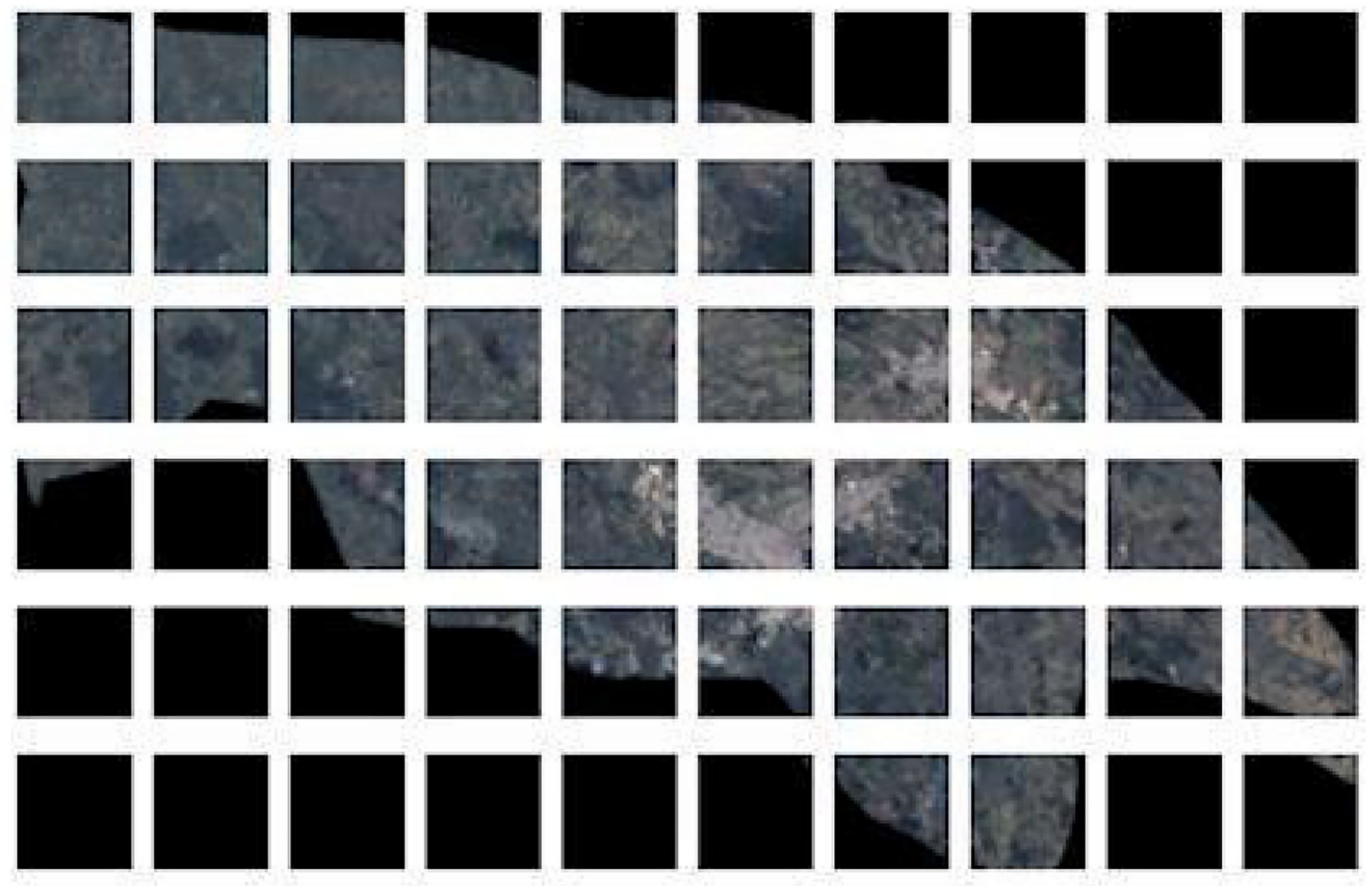
Patch generation. Satellite image of municipality of Popayán divided into patches of images of 256 × 256 pixels used to generate the predictions of features found in each patch.

Once the model was selected, we made predictions on the dataset of images extracted from GEE. As an image of a municipality is usually much larger than the images in which the model was trained, this image must be divided into 256 × 256 pixel patches, as seen in Figure 4. In Figure 4, it is also noticed that the mask (black region) covers all or most of the patch. For this reason, only patches with relevant information were selected. To select the patches with relevant information, a threshold of 30% was defined for the maximum number of clouds (totally white pixels) and mask (totally black pixels) admitted in each patch. If a patch does not contain at least 70% of the terrain information, it was removed.

With the 256 × 256 patches, the prediction was made using the ResNet 50 model on each patch. This told us whether there is a presence of the classes in each image or not. With the results of these predictions, an estimate was calculated for the number of water sources, crops, populated areas, or roads in a specific department or municipality.

#### Data Preparation for Metadata

- Nutrition data and Agricultural Census: the data from these sources contains many variables, therefore we just selected those directly related to the GFSI, ensuring that the data was numeric data or binary fields (yes/no) to facilitate its processing. In the selected variables the “no” values were assigned to 0 and “yes” values to 1. Also, within the data cleaning process, the empty data was replaced with 0 so that it did not have any weight in the final count when we were counting the proportion of yes values over the total. The rows with missing data with other numerical values were dropped, except in the case of the vitamins table, there were many variables with empty data. In this case, we decided to impute the missing data with the average of the corresponding variable. Once the data was cleaned, a grouping of each table was made concerning the key variable that relates all the tables. Next, all the tables were grouped by the municipality to have a single value for each column per municipality adding the number of ones (yes) and dividing over the total of answers to have the proportion of yes.
- Health Records: health data provided information on hospital admissions. Therefore, they have data irrelevant for the GFSI. For this reason, the fields that had to do only with nutrition were filtered, and a dataset was generated where the rows are the municipalities, and the columns are the number of reports in a specific week in chronological order.
- Meteorological Data: this data was extracted from raster files containing the pixel value (temperature or precipitation in a specific month) and the position (latitude and longitude). Therefore, having the geographic coordinates of a location, these data was extracted at that point to build a dataset.

Finally, all the nutrition, agriculture, health, and meteorological data were integrated into a single dataset, joining them by a municipality code. As a result, the dataset obtained contains 926 columns, representing different multidimensional characteristics, and 9 rows, each row representing a municipality of Department of Cauca where data could be obtained (Popayán, Balboa, Bolívar, Paez, Patía, San Sebastián, Santander de Quilichao, Timbío, Timbiquí). Although not all columns were used to calculate the GFSI, having so many characteristics allows this dataset to be used for further research.

A dataset with all 42 municipalities of Cauca was also generated. However, due to the lack of nutritional data in several municipalities, the data of the missing municipalities must be imputed in order to calculate the GFSI for all these municipalities. A simple solution to solve this problem of lack of data was by imputing in the empty variables the average value for the 9 municipalities with nutritional data. However, for simplicity and quality of the results, the rest of the article will continue to be explained only for the dataset with 9 municipalities.

### Modeling and Evaluation

In the modeling and evaluation stage, a model to calculate the GFSI was built. Here it is important to bear in mind that the evaluation of the model is not done with the same data in which the model was trained, since the model could be memorizing these data and we expect it to generalize to other datasets.

For the construction of the model, the data available in the GFSI in 2020 by country, published annually by the EIU (3), was used. This data comprises 60 columns or items and 113 rows, each row representing a country. It should be noted that these data were not normalized, therefore for each variable, the normalization indicated by the GFSI was carried out to convert the variables into a range from 0 to 100.

With the normalized dataset of GFSI in 2020 by country, a variable to predict was defined, this was the GFSI score for each country, and the other 59 variables will be the data used to train the model to predict the GFSI. The dataset was randomly divided into training and testing using 80% of the data for training and the remaining 20% for testing.

The metric chosen to evaluate the model was the Mean Absolute Error (MAE). Different models were tested on the normalized and non-normalized dataset of GFSI in 2020 by country (see Table 1). We obtained better performance with linear regression in both cases.

**Table 1:** GFSI score by the municipality.

**Model**	**MAE (normalized)**	**MAE (not normalized)**
Linear regression	0.391	2.714
Ridge regression	0.391	3.338
Lasso	0.408	3.442
Elastic net	0.398	3.423
Multilayer perceptron	0.825	4.383
Support vector machine	0.379	3.542
Random forest	3.286	3.315

*The table shows the mean absolute error of the models tested for the calculation of the GFSI using a test portion of the GFSI dataset by country provided by the EIU, the evaluation was done with normalization and without normalization*.

Once we have the best model, we calculated the food security index in the municipalities of the Department of Cauca for which the dataset was built. To calculate the GFSI, we tried to approximate the required variables with the variables available in the created dataset. In this way, it was possible to reconstruct the GFSI on a departmental or even municipal scale.

For some GFSI items, there were no variables at the municipal level in the dataset created, so variables at the departmental or national level were used. Some of them were taken from the GFSI of Colombia for the year 2014.

## Results

The results described in this article are divided into the same two sections according to the two existing data modalities, satellite images, and metadata for calculating the GFSI.

### Feature Extraction From Satellite Images

Due to the model task, which is a multi-label classification task, the selected metrics were accuracy, precision, recall, and F1-score. With these metrics, it is possible to see the model's performance in each characteristic. Furthermore, we can also see a sample of the model's performance globally using the macro-accuracy, macro-precision, macro-recall, and macro-F1-score, which is an average of the performance of a model for each feature.

**Figure 5:**
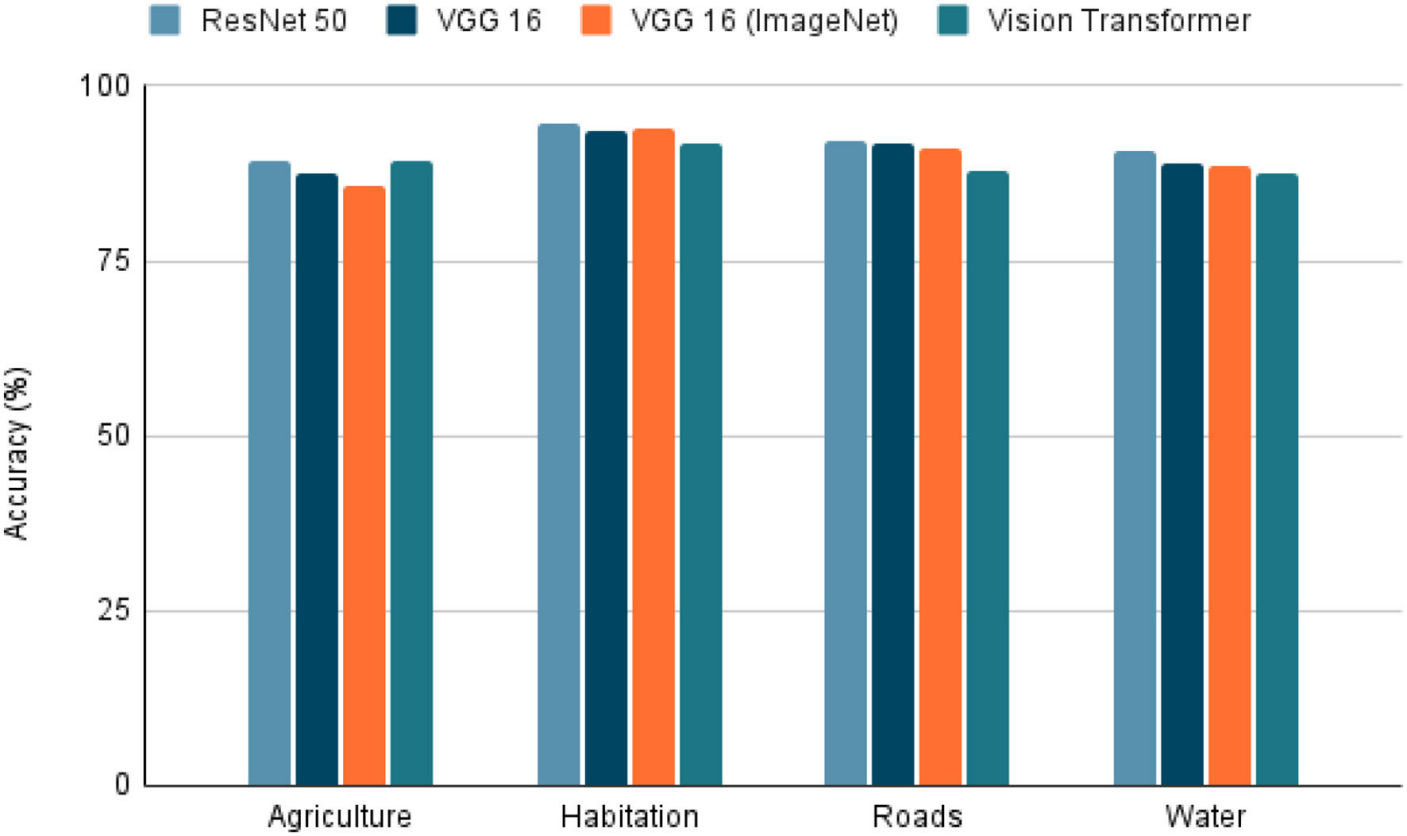
Accuracy on tested models. Accuracy of the 4 models tested in the extraction of the 4 characteristics (agriculture, habitation, roads, whater). A highest value indicates a better performance in the classification task.

**Figure 6:**
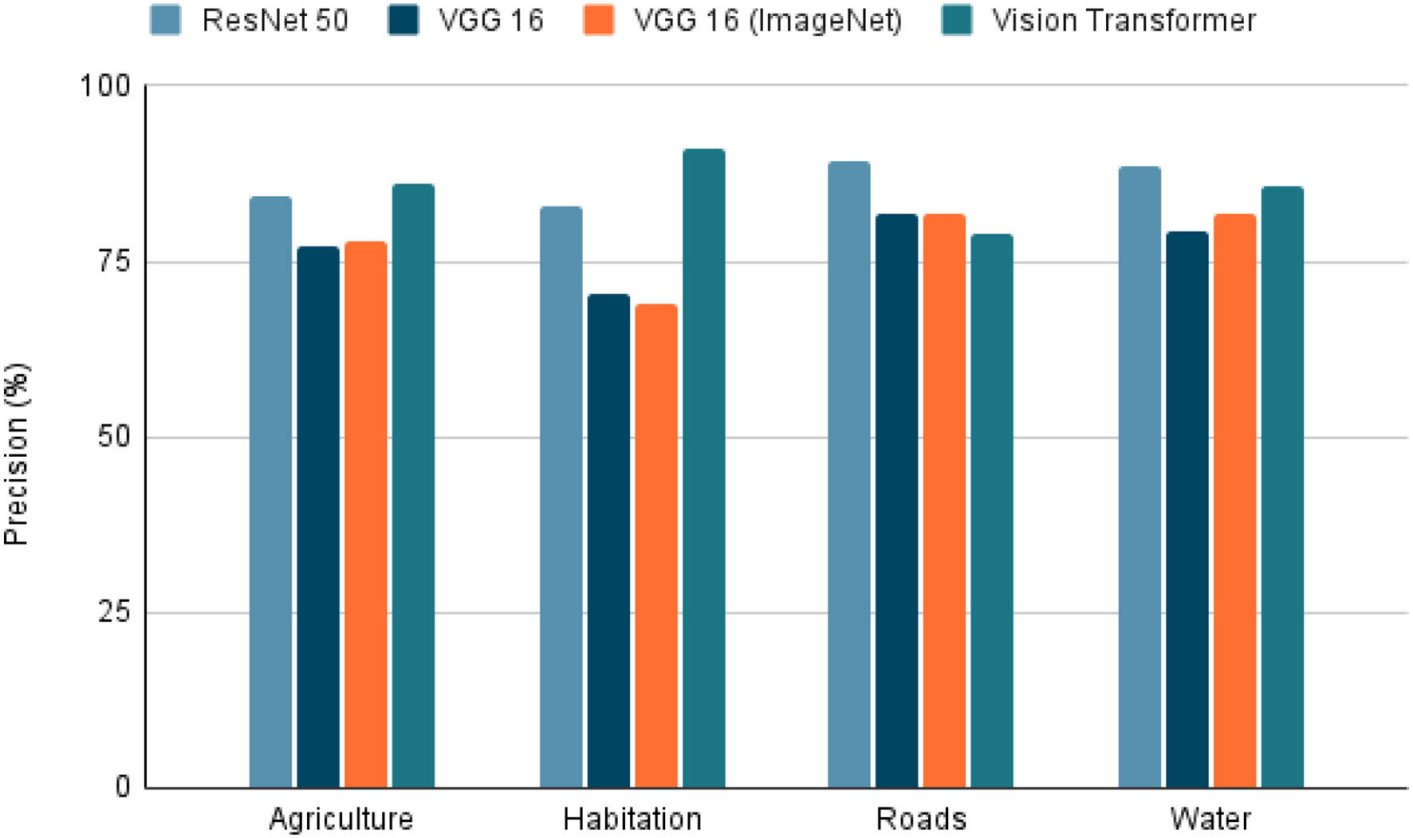
Precision on tested models. Precision of the 4 models tested in the extraction of the 4 characteristics (agriculture, habitation, roads, whater). A highest value indicates a better performance in the classification task.

**Figure 7:**
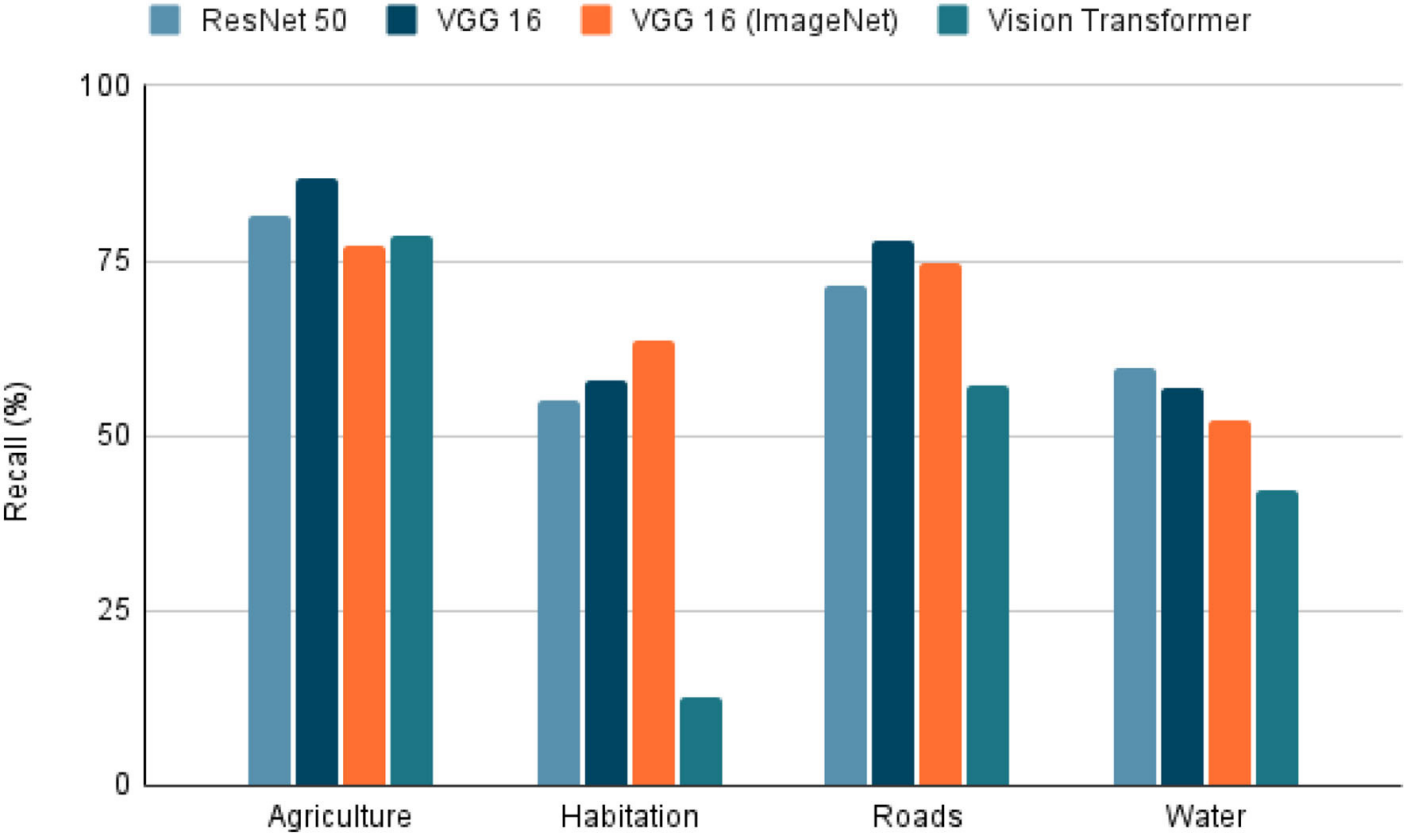
Recall on tested models. Recall of the 4 models tested in the extraction of the 4 characteristics (agriculture, habitation, roads, whater). A highest value indicates a better performance in the classification task.

**Figure 8:**
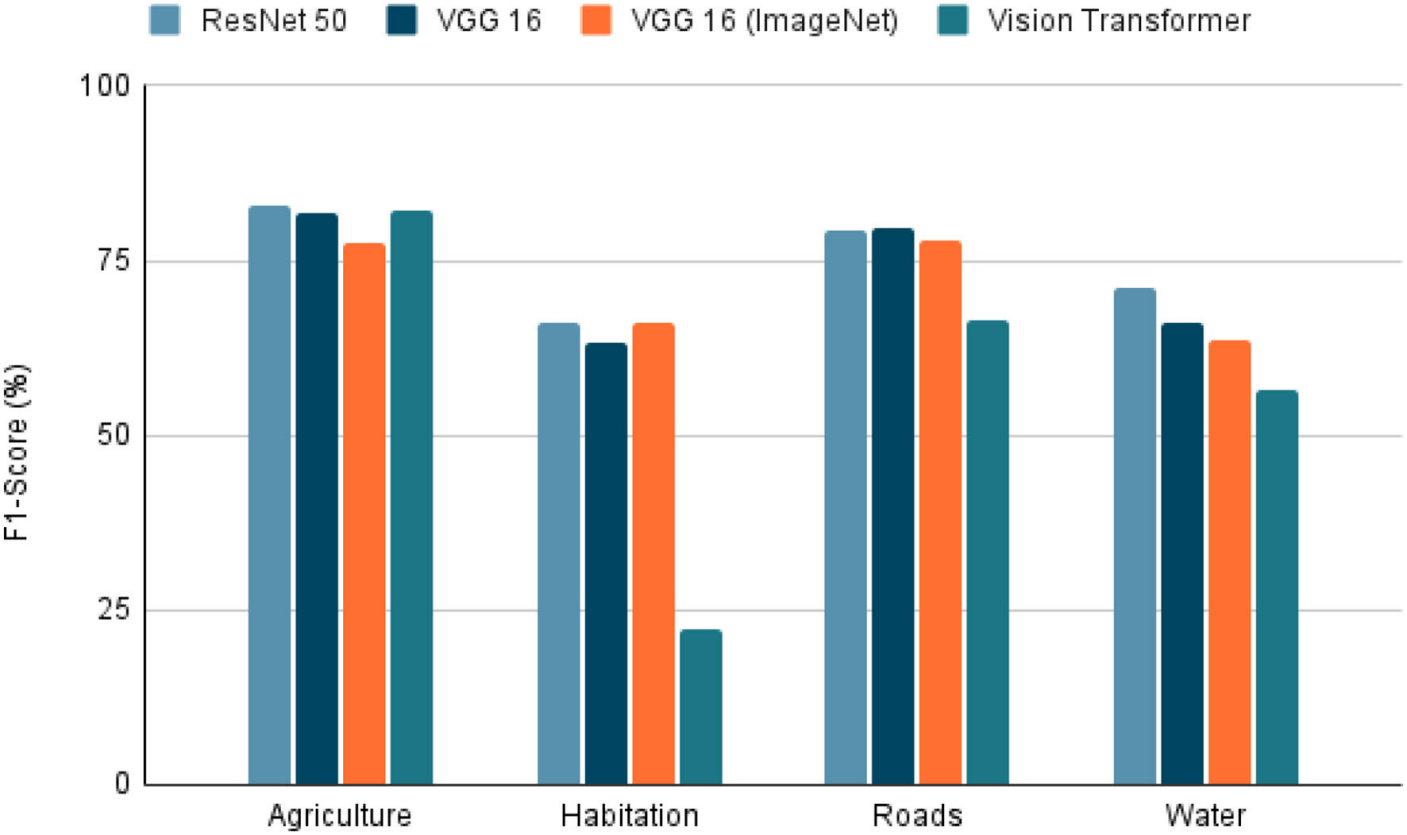
F1-score on tested models. F1-score of the 4 models tested in the extraction of the 4 characteristics (agriculture, habitation, roads, whater). A highest value indicates a better performance in the classification task.

Within the tested models, the accuracy, precision, recall, and F1-score results are presented in Figures 5–8. The F1-score is selected as the main metric because it is the most balanced one. As a result, the architecture of the Convolutional Neural Network (CNN) ResNet 50 has better performance in most tasks. Therefore, it can be said that the model performs well on most labeled data, even with unknown data. However, these metrics could decrease since the images used for the prediction extracted from Landsat-8 have different resolution and zoom levels than the images in the dataset the model was trained with and evaluated. The reason for selecting images from a different source than the one used in Kaggle, is to ensure that the experiment can be reproduced. Furthermore, the use of Landsat-8 images is open and can be done anywhere in the world.

**Figure 9:**
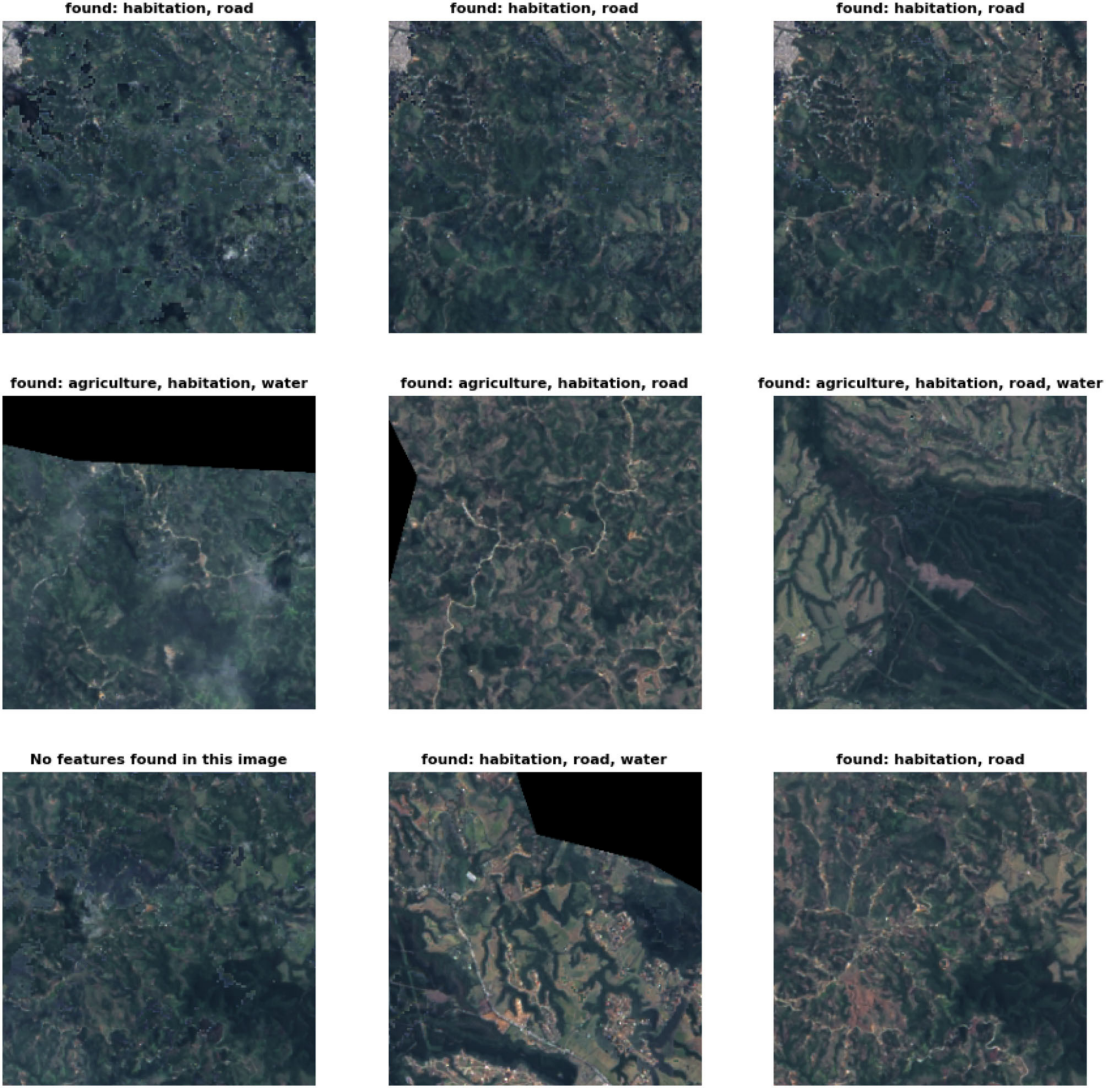
Model predictions over Popayán patches. Examples of predictions generated over some patches of Popayán using the ResNet 50 model with highest f1-score in extraction of 4 features from satellite images. Above each patch are the characteristics that were found or if none of the 4 characteristics were found, it is indicated that no characteristics were found in that image.

Finally, the predictions of the characteristics on the 256 x 256 patches for the municipality of Popayán in Cauca can be seen in Figure 9. Each image is labeled to obtain the presence of the four characteristics with which the model was trained with.

### Calculation of the GFSI for the Department of Cauca

The results obtained concerning the GFSI for the Department of Cauca, both for the model trained with the normalized and non-normalized datasets of GFSI at a country level, are found in Table 2. There is a significant difference between normalized and unnormalized data (*p* = 0.15719). Here we see how the Department of Cauca with an index of 57.444 calculated with normalized data, presents a deficit in food security (−5.756 points) concerning the value of the GFSI of Colombia which is 63.2 for the year in which the index was calculated by EIU. The score for the Department of Cauca, which is made up of 42 municipalities, is obtained through the average of the scores of the nine municipalities (Popayán, Balboa, Bolívar, Paez, Patía, San Sebastián, Santander de Quilichao, Timbío, Timbiquí) for which data are available. According to the results, it can be seen that the municipality with a lower food security index is Timbiquí, which has a value of 53.637 with normalized data. When compared with the index calculated for the Department of Cauca which is 57.444 (normalized data), this municipality presents a variation of 3.807 points below the average. In addition, it is observed that this municipality has 9.563 points (normalized) below the national GFSI for 2014.

**Table 2:** GFSI score by municipality.

**Municipality**	**Unnormalized GFSI**	**Normalized GFSI**
Popayán	59.500	60.569
Balboa	54.900	57.019
Bolívar	55.583	56.787
Paez (Belalcázar)	56.112	57.741
Patía (El Bordo)	55.052	56.722
San Sebastián	55.857	56.821
Santander de Quilichao	57.812	59.856
Timbío	57.373	58.444
Timbiquí	53.219	53.637
Cauca	56.156	57.444

*The table shows the value of the GFSI score of 9 municipalities of Cauca using both normalized and unnormalized data. The index was calculated using the models with best performance in both the normalized and unnormalized datasets of the GFSI by country by the EIU used for training*.

The municipality of Popayán is the one that obtained the best result in the Department, with scores of 60.569 (normalized). When comparing it with the result of the whole department, a difference of 3.125 points (normalized) is observed above the average. This is an expected value since Popayán is the capital city, while the other municipalities might receive less attention by the national and departmental government. However, although comparing the result with the average value of Colombia, we obtained 2.631 points with normalized data, below the national average.

## Discussion

This article presents the process of building a multi-dimensional dataset of open data and satellite images to characterize food security in the Department of Cauca, Colombia. The dataset is structured according to the four-dimensional model of food security proposed by FAO (2). It allows the calculation of a multidimensional food security index. This research presents the calculation of the index for nine municipalities in the Department of Cauca, using the GFSI model established by the EIU (3).

Likewise, as an alternative to the lack of some data, the option of using satellite images is presented. A method is proposed to extract characteristics from these and integrate them into a dataset that includes metadata as well. Only four characteristics were extracted (Agriculture, Habitation, Roads, and / or Water). However, this same method can be used to extract other types of characteristics. The characteristics extracted can be used for the dimensions of physical availability (Agriculture, Water) and physical access to food (Roads, Habitation), and sustainability over time, due to the temporal resolution that is better than most surveys and censuses.

The data obtained came from various sources, which differed from the GFSI calculation during their collection. Therefore, an arduous pre-processing stage was required, and data transformations were consistent with those used by the GFSI. This includes data mining, imputation techniques, operations between dataset variables, changes in scales, units, etc. Also, in some cases, the variables for the GFSI calculation were not found within the created dataset, so the models were developed using also data manually imputed in the dataset obtained from reports produced by local, departmental or municipal governments.

The use of the CRISP-DM methodology for the construction of the dataset and calculation of the index gives us a framework for the project phases, from the understanding of the problem to the validation of the results. Furthermore, the CRISP-DM methodology allows us to correct errors promptly and return to early phases of the project. In addition, the stages are appropriate to facing in data mining projects, as it presents a logical order and good practices.

About the model performance, as can be seen in the results regarding the model for calculating the GFSI trained with the dataset provided by the EIU of the GFSI at country scale (Table 1), simpler models have better performance than more complex models such as the multilayer perceptron or support vector machines (SVM). Also the Normalization decrease the MAE. These results may occur due to the nature of the data, because the GFSI is usually calculated by a group of experts assigning weights to each of the 59 variables, therefore the linear models such as linear regression should have a good performance. Another reason is the number of columns in relation to the instances (number of countries used in the GFSI). Having so few instances and many columns can cause overfitting in more complex models so they will not generalize well to new data, this problem can be solved by adding the GFSI by country data in other years to the actual dataset used to train the model.

The obtained results reveal a possible food security problem in the Department of Cauca. Historically, this department has been affected by violence, armed conflict, drug trafficking, and corruption, that determine the distribution of equitable resources (37). Therefore, the national government could take this index as a variable to consider in future decision-making in the country, seeking to improve the food security situation of the department and the municipalities that comprise it.

Although the GFSI calculation seeks to identify the food security situation for the Department of Cauca and some of its municipalities, this model can calculate the index in other departments, municipalities, or countries. Therefore, the same process could be followed to build the dataset and ML model with data from the place to be studied.

The other works carried out in the field of food security that makes use of ML focus only on one dimension defined by FAO (9–24), more specifically on the dimension of physical availability of food (primarily focused on predicting crop yield). So it would not give us a total vision of food security problems according to what the FAO proposes. Furthermore, in no case did the studies related to ML in food security calculate an index (9–24). In the studies not using ML techniques to calculate a food security index on a specific regional scale, indices other than GFSI are generally used, not covering the four dimensions FAO proposed (5, 6). This is probably due to the difficulties involved in collecting the data necessary for calculating the GFSI at such a specific level.

Although the methodology has been shown to comply with the work when creating the dataset and calculating the GFSI, some limitations arise in terms of the availability and nature of the data and must be taken into account. Many data are qualitative and must be calculated by experts in the area and other is difficult to find at the municipal and / or departmental level. Given this, the solution to cover these data may be to impute the data used in the GFSI at the country level. Consequently, the index obtained can be biased by external national factors that do not necessarily reflect the food security situation in the department or municipality.

Another limitation of the model is temporality. The data, in some cases, is released with a long delay. This produces results based on outdated data. Also, the frequency with which much of the data is collected is not desired since data is collected every 5 years or more in some cases (i.e., census). This is not enough to support the fourth dimension of FAO, which is stable over time.

In addition, the data are also incomplete on a spatial scale. Some surveys, such as ENSIN, are carried out only for 9 of the 42 municipalities of Cauca. Therefore, a GFSI calculation cannot be obtained with such precision for all the municipalities of Cauca due to the lack of data. This deficiency could be covered by satellite images that have spatial and temporal scale, and are openly accessible to any government, academic institution or civil society. However, the use of these images increases the computational resources required for data processing.

Nevertheless, one limitation for the satellite images used here is that the dataset used to train the model was not created with images from the same satellite, which could add noise to the predictions. This problem could be solved using satellite images from the same satellite and resolution as the Kaggle Planet dataset. Also, using another satellite with higher resolution, such as Sentinel-2, could improve the predictions, although it should be noted that this is only available from 2015. Another alternative could be to use a dataset made with Landsat-8 images to train the model, but it is essential to remember that this will require time and expertise.

## Conclusion

This paper proposes the methodology for creating an open dataset combining machine learning techniques to calculate a food security index at a departmental or municipal level, covering the four dimensions described by FAO. Data from different open sources such as censuses, surveys, health records, and satellite images are used to create the dataset, giving an economical alternative and easily accessible to all governments, academic institutions and civil society. It shows how the CRISP-DM methodology can be used to create an open public health data repository. Furthermore, this methodology could be generalized to other types of problems requiring the creation of a dataset. As well as the use of satellite images which presents an alternative for places where data collection is challenging, either for economic or access reasons.

In addition, a dataset with more variables than those used in the GFSI is released, opening research opportunities to areas like nutrition, agriculture, climate change, among others, where problems different to the GFSI and / or the individual dimensions of FAO could be analyzed.

As future work, the investigation of new models and methods for the feature extraction of satellite images is proposed. Also, the quality of the dataset and predictions could be improved by using more bands in satellite images than RGB or with newer sources of satellite images such as sentinel-2 or sentinel-3, as well as novel preprocessing methods due to increased spatial and temporal resolution in the images.

## Data Availability Statement

Datasets has been submitted to Mendeley data. The datasets generated and used for this study can be found with the name “Multidimensional Dataset Of Food Security And Nutrition In Cauca” in Mendeley data at this link: https://data.mendeley.com/datasets/wsss65c885/draft?a=da27eda0-9529-4ad8-9922-6ffe8de79756.

## Author Contributions

DL and RV-C contributed to conception and design of the study, as well as in the constant advice during the project. DL contributed in the data identification and acquisition. LP and DR contributed in the data analysis and preprocessing of metadata. DR contributed to the acquisition of satellite images, their preprocessing and the modeling stages using machine learning. JO-V in the review of work and theoretical contributions from the point of view of public health. All authors contributed to manuscript revision, read, and approved the submitted version.

## Conflict of Interest

The authors declare that the research was conducted in the absence of any commercial or financial relationships that could be construed as a potential conflict of interest.

## Publisher's Note

All claims expressed in this article are solely those of the authors and do not necessarily represent those of their affiliated organizations, or those of the publisher, the editors and the reviewers. Any product that may be evaluated in this article, or claim that may be made by its manufacturer, is not guaranteed or endorsed by the publisher.
